# Improvements of Paraquat Treatment in Liquid Media for Behavior and Neurodegenerative Tests

**DOI:** 10.17912/micropub.biology.001273

**Published:** 2025-03-29

**Authors:** Axel J. Ufarry Alvarado, Malik A. Zaidi Pons, Jayvier Plaza Hernández, Ceidy Torres Ortiz

**Affiliations:** 1 Deparment of Biomedical Sciences , Pontifical Catholic University of Puerto Rico, Ponce, Puerto Rico; 2 Deparment of Biomedical Sciences, Pontifical Catholic University of Puerto Rico, Ponce, Puerto Rico; 3 Deparment of Natural Sciences, Pontifical Catholic University of Puerto Rico, Ponce, Puerto Rico

## Abstract

Amyotrophic Lateral Sclerosis (ALS) is a disease characterized by misfolded and aggregated proteins that have toxic effects on motor neurons. The missense mutation, G85R, of the *sod-1* gene associated with ALS displays locomotor impairments in *Caenorhadbitis elegans* ( *C. elegans* ). We treated the *sod-1 (G85R)* strain with 0 and 2.5 mM Paraquat treatments in a liquid M9 buffer for 4 hours and in solid NGM media for 18 hours. In both methodologies, the locomotion defects and neurodegeneration were significantly increased with 2.5 mM Paraquat. Our work provides evidence of methodology that is more cost effective, rapid and reproducible to perform behavior and neurodegenerative assay in worms.

**Figure 1. Behavior and Neurodegeneration assays conducted with sod-1(G85R) strain:**
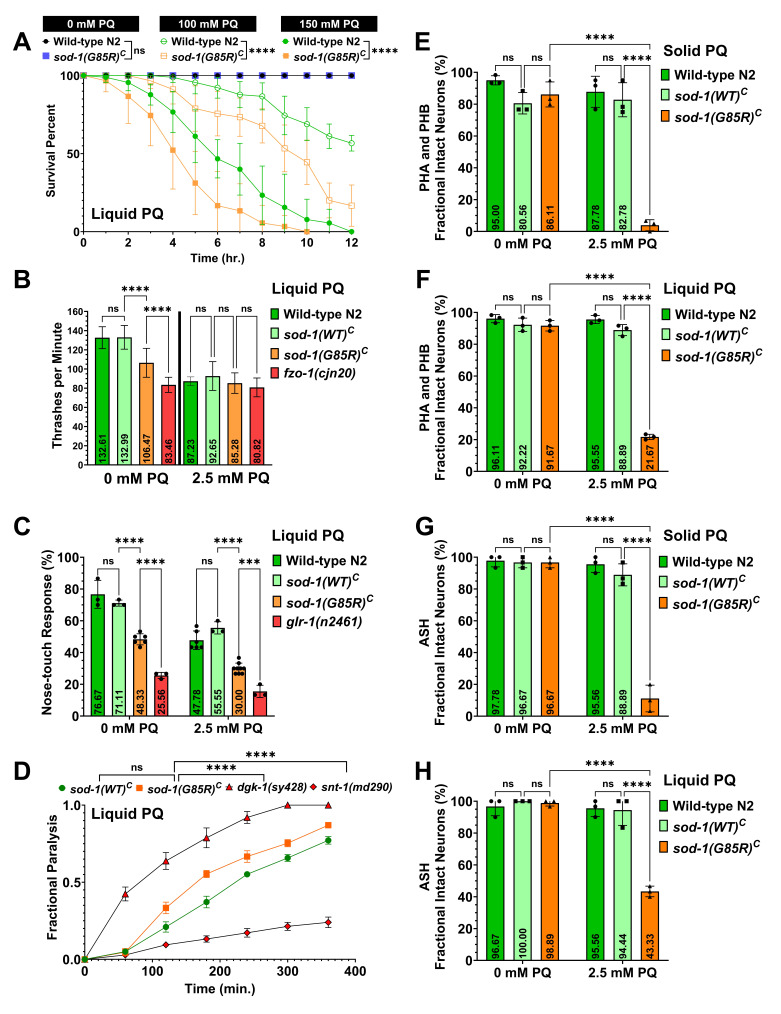
**(A)** Survival of worms treated with 100 and 150 mM Paraquat (PQ) in liquid media. The *sod-1(G85R) ^C^* strain showed a significant decrease in survival with PQ treatment compared to *sod-1(WT) ^C^* . Three independent experiments with three replicates (n=90 worms per group) were analyzed. A log rank test is used with DF=1, *p* <0.0001. Error bars indicate standard deviation for each time. **(B)** Quantification of number of thrashes per minute of worms treated with 0 and 2.5 mM Paraquat (PQ) in liquid media. The *sod-1(G85R) ^C^* strain has defects in locomotion that increased with PQ treatment compared to s *od-1(WT) ^C^* . Three to six independent experiments with 10 worms per group were analyzed using a One-way ANOVA with F=152.2 and *p* <0.0001. Significance on the graph: * *p* = 0.0319; **** *p* <0.0001. Error bars indicate standard deviation. **(C)** Nose touch avoidance response of worms treated with 0 and 2.5 mM Paraquat (PQ) in liquid media. The *sod-1(G85R) ^C^* strain has a defect in nose touch avoidance response and the defect increased with PQ treatment compared to *sod-1(WT) ^C^* . Three to six independent experiments with 30 worms per group were analyzed using a Two-way ANOVA with *p* <0.0001 for genotype, *p* <0.0001 for treatment and *p* =0.0007 for interaction between genotype and treatment. Error bars indicate standard deviation. **(D)** Aldicarb resistance assay. No difference was observed between *sod-1(WT) ^C^* and *sod-1(G85R) ^C^* . The number of independent experiments were 7 for *sod-1(WT) ^C^* strain, 3 for *sod-1(G85R) ^C^* strain and 5 for *dgk-1 and snt-1* strains with 15 worms per group. Seven to three independent experiments were analyzed using a log rank test with DF=1, *p* <0.0639 for *sod-1(WT) ^C^* and *sod-1(G85R) ^C^* , *p* <0.0001 for *sod-1(G85R) ^C^* and *dgk-1* ; *sod-1(G85R) ^C^* and *snt-1* . Error bars indicate standard deviation. **(E-H)** Dil staining assay in worms exposed at 0 and 2.5 mM of PQ in **(E, G)** solid for 18 hours and in **(F, H)** liquid media for 4 hours. The *sod-1(G85R) ^C^* strain has neurodegeneration of ASH, PHA and PHA neurons with PQ treatment regardless of solid or liquid medium. Three independent trials with 15 worms per group were analyzed using Two-way ANOVA with *p* <0.0001 for genotype, *p* <0.0001 for Paraquat treatment and *p* <0.0001 for interaction between genotype and PQ treatment for both solid and liquid experiments. Error bars indicate standard deviation.

## Description

Amyotrophic Lateral Sclerosis (ALS) is a motor neuron disease characterized by the loss of cholinergic and glutaminergic neurons. Although the cause of ALS is unknown, the *Sod1* gene was one of the first genes related to the disease (Rosen et al., 1993; Nguyen, 2024). The *Sod1* gene codifies for Cu/Zn superoxide dismutase 1 ( SOD-1 protein, which is an antioxidant enzyme catalyzing the conversion of superoxide ions (O _2_^-^ ) to hydrogen peroxide (H _2_ O _2_ ). Hundreds of missense mutations have been identified in the *SOD1* gene that are associated with ALS (Mathis et al., 2019; Huai & Zhang, 2019 and Abel et al., 2012). Some SOD1 mutations have a loss and toxic gain of function that contributes to ALS progression (Qualls et al., 2013; Sahin et al., 2017; Baskoylu et al *.,* 2018).

*C. elegans* has served as a model for ALS, with *sod-1* mutations conferring sensitivity to oxidative stress including Paraquat treatment (Osborne et al., 2021; Wang et al., 2009). A previous study by Baskoylu et al. (2018) reported that *C. elegans* single copy/knock-in model for G85R was associated with cholinergic and glutamatergic neurodegeneration after oxidative stress. In the current study, we asked if similar results could be observed when experiments are conducted in liquid media (M9 buffer) instead of solid media (nematode growth medium, NGM).

To this extent, we used the *sod-1 (G85R) ^C^* strain, as well as the *sod-1 (WT) ^C^* and N2 wild type strains. The *sod-1 (WT) ^C^* strain contains all the silent codon changes needed for CRISPR/Cas9 genome editing of the *sod-1 (G85R) ^C^* strain (Baskoylu et al., 2018). First, we tested the nematode survival rate under PQ-induced oxidative stress. The *sod-1 (G85R) ^C^* strain and N2 wild type were exposed to 0, 100 and 150 mM PQ for 12 hours. Results showed **(Fig A)** , that at the highest PQ concentration, the *sod-1 (G85R) ^C^* mutants survival rate had decreased survival compared to wild type. The liquid media by itself did not have an effect on survival. Then we tested if the locomotion alterations associated with cholinergic and glutamatergic defects in *sod-1 (G85R) ^C^* strain could be replicated in liquid media. The nose touch avoidance assay was used to evaluate the response of glutamatergic neurons such as ASH (Davis et al., 2022) and the thrashing assay to evaluate locomotion mediated by cholinergic motor neurons and muscle cells. The results showed a decrease in thrashing in the absence of PQ in the *sod-1 (G85R) ^C^* strain when compared to controls strains, and a defective nose touch avoidance response with or without PQ-induced oxidative stress **(Fig B & C)** . Even though the *sod-1 (G85R) ^C^* strain showed locomotion defects, no changes in synaptic transmission of cholinergic neurons were observed **(Fig D)** . Nevertheless, the aldicarb resistance assay showed accelerated paralysis in *sod-1 (G85R) ^C^* strain when compared to wild type, with a critical point at 180 minutes, indicating defective neuromuscular signaling. Since defects on nose touch avoidance response assay may be associated with glutamatergic neurodegeneration, we compared the uptake of a lipophilic fluorescent dye under PQ treatment in solid vs. liquid media. The *sod-1 (G85R) ^C^* strain showed a decrease in the percentage of intact ASH, PHA and PHB neurons in both media **(Fig E-H)** . However, these effects were observed earlier during PQ exposure in liquid media (4 hr, **Fig F & H** ) compared to solid media (18 hr, **Fig E & G** ).

Overall, we were able to consistently reproduce the results observed in the *sod-1 (G85R) ^C^* single copy/knock-in C. elegans model developed by Baskoylu et al. (2018) regardless of whether treatment is done on solid or liquid media. Therefore, we conclude that it is possible to conduct studies of neurodegeneration and behavior in *C. elegans* using liquid media, which allows it to be conducted in less time and at lower cost.

## Methods

**Worm maintenance:** The Nematode Growth Medium (NGM) [17 g agar, 3 g NaCl, 2.5 g peptone, 1 ml 1M CaCl _2_ , 1 ml 5 mg/ml cholesterol in ethanol, 1 ml 1 M MgSO _4_ , and 25 ml of 1M KPO _4_ ] plates were seeded *with Escherichia coli* OP50 and incubated to 37°C overnight and stored at room temperature in boxes or used for cultivating the worms. The worms were transferred every 3-4 days to seeded plates with *Escherichia coli* OP50 . All worm strains were maintained in an incubator at 20°C.

**Worm synchronization:** Young adult worms were transferred to NGM plates and incubated at 20°C until the next day. After removing the young adult worms, the eggs remain on the plate incubated for four days at 20°C to let the worms reach day 1 of adulthood. For all the experiments the worms were synchronized to the first day of adulthood.

**Paraquat treatment in liquid and solid media:** The worms were transferred to a well (24-well plate) or microtubes with M9 buffer. Then, the treatments were added to obtain a final concentration Paraquat in M9 buffer (refers to PQ Liquid) for 4 hours. After incubation worms were transferred to solid plates with *E.coli* OP50 for 30 minutes before experimental assay. For solid PQ, worms were transferred to NGM plates seeded with *E. coli* OP50 with 0 mM and 2.5 mM Paraquat (refers to PQ solid) for 18 hours. After incubation worms were transferred to solid plates only with *E.coli* OP50 for 30 minutes before experimental assay. All experiments were conducted blinded for genotype and treatment.

**Survival test:** The worms were transferred to a well (24-well plate) with 100 or 150 mM Paraquat treatment diluted in M9 buffer (refers to PQ liquid). The worm was counted dead if no movement occurred after touching the worms 3 times in the head and tail consecutively with a platinum wire. The worms were evaluated each hour for 12 hours. The survival percentage was calculated and graphed with the standard deviation of the three independent experiments (N=3).

**Nose Touch Avoidance Assay:** The evaluation consists of placing a single brush bristle perpendicular to the front of a worm moving forward, then waiting for a nose collision. If the worm responds with reverse locomotion, it is classified as non-defective. The nose touch avoidance percentage was calculated of the total non-defective worms divided by the total number of worms evaluated multiplied by 100. The nose touch avoidance percentage was calculated and graphed with the standard deviation of the three independent experiments (N=3).

**Thrashing assay:** The worms were transferred to a well with 50 µL of M9 buffer in a 96-well plate. The worms were left for 30 seconds to acclimatize in the well. A three-minute video of the worms swimming was taken with a NIKON SMZ745T stereoscope with Kopa Software. The evaluation consists of processing the video in slow motion and counting the number of thrashes (C-shape) per minute. An average of three minutes was calculated for each worm. The number of thrashes was calculated and graphed with the standard deviation of the three independent experiments (N=3).

**Aldicarb Resistance Assay:** The worms were transferred to a microtube with M9 buffer for 4 hours at 20°C. The worms were transferred to the NGM plates with 1 mM aldicarb seeded with *E. coli* OP50 and evaluated for worm paralysis each hour for 6 hours. Paralysis was defined as the absence of movement and pumping for 5 seconds after touching the worm head and tail 3 times consecutively. The fractional paralysis was calculated and graphed with the standard deviation of the three independent experiments (N=3).

**DiI staining assay:** After treatment, the worms were washed and incubated with ('DiI'; DiIC 18 (3)) D282 for 2 hours. All worms were transferred to NGM plates seeded with *E. coli* OP50 for 30 min. Up to 15-20 worms placed in microscope slides were immobilized with 10 mM levamisole. The ASH in the head and the PHA and PHB in the tail were visualized at 575-625 nm and scored. The percentage of fractional intact neurons was calculated and analyzed in the three independent experiments (N=3).

**Statistical analysis**

Quantitative data for the statistical analysis is organized and processed using the Microsoft Office 2011 Excel software package (Microsoft Corporation, Redmond, USA) and analyzed using GraphPad Prism version 10.0.0 for Windows (GraphPad Software, Boston, Massachusetts, USA). The standard deviation is used to present the maximum and minimum values with the meaning in graphs. The value of *p* < 0.05 is considered statistically significant. For nose touch avoidance and neurodegeneration assays a Two-way ANOVA was used for statistical analysis. For thrashing assay, a One-way ANOVA was used for statistical analysis. For survival and aldicarb resistance assays a log rank test is used to compare distribution of time of curves and determine significant differences.

## Reagents

**Worm and bacteria strains**

The following strains were obtained from the Caenorhabditis Genetics Center (CGC) funded by NIH Office of Research Infrastructure Programs (P40 OD010440)

**Table:** 

**Strain**	**Genotype**	**Available from**
N2	*C. elegans* wild isolate.	CGC
HA2986	sod-1 ( rt448 [ sod-1 WT C]) II	CGC
HA3299	sod-1 ( rt451 [ sod-1 G85R]) II	Dr. Hart Lab, Bio Med Center Brown University
KP4	glr-1 ( n2461 ) III	CGC
BXN723	fzo-1 ( cjn20 ) I	CGC
NM204	snt-1 ( md290 ) II	CGC
PS2627	dgk-1 ( sy428 ) X	CGC
*Escherichia coli*	OP50	Dr. Colon Ramos Lab in Yale University
